# Kairos study protocol: a multidisciplinary approach to the study of school timing and its effects on health, well-being and students’ performance

**DOI:** 10.3389/fpubh.2024.1336028

**Published:** 2024-03-08

**Authors:** Daniel Gabaldón-Estevan, Diego Carmona-Talavera, Belén Catalán-Gregori, Elena Mañas-García, Vanessa Martin-Carbonell, Lucía Monfort, Elvira Martinez-Besteiro, Mònica González-Carrasco, María Jesús Hernández-Jiménez, Kadri Täht, Marta Talavera, Ana Ancheta-Arrabal, Guillermo Sáez, Nuria Estany, Gonzalo Pin-Arboledas, Catia Reis

**Affiliations:** ^1^Department of Sociology and Social Anthropology, University of Valencia, Valencia, Spain; ^2^Service of Clinical Analysis, University Hospital Dr. Peset, Valencia, Spain; ^3^Department of Education, Valencian International University, Valencia, Spain; ^4^Department of Pediatrics, Dr. Peset University Hospital, Valencia, Spain; ^5^Department of Pediatrics, Clinical University Hospital, Valencia, Spain; ^6^Department of Personality, Assessment and Psychological Treatments, University of Valencia, Valencia, Spain; ^7^Research Institute on Quality of Life, University of Girona, Girona, Spain; ^8^Faculty of Health Sciences, Valencian International University, Valencia, Spain; ^9^Institute of International Social Studies, School of Governance, Law and Society, Tallinn University, Tallinn, Estonia; ^10^Department of Experimental and Social Sciences Teaching, University of Valencia, Valencia, Spain; ^11^Department of Comparative Education and History of Education, University of Valencia, Valencia, Spain; ^12^Department of Biochemistry and Molecular Biology, Faculty of Medicine and Dentistry, University of Valencia, Valencia, Spain; ^13^Grupo de Sueño y Cronobiologia de la Asociación Española de Pediatría, Valencia, Spain; ^14^CRC-W - Faculdade de Ciências Humanas, Universidade Católica Portuguesa, Lisbon, Portugal; ^15^Instituto de Medicina Molecular João Lobo Antunes, IMM, Lisboa, Lisbon, Portugal; ^16^ISAMB - Faculdade de Medicina Universidade de Lisboa, Lisbon, Portugal

**Keywords:** childhood, adolescence, circadian rhythms, chronotype, school schedule, social jetlag, well-being

## Abstract

Recent evidence from chronobiology, chssronomedicine and chronopsychology shows that the organisation of social time (e.g., school schedules) generally does not respect biological time. This raises concerns about the impact of the constant mismatch between students’ social and internal body clocks on their health, well-being and academic performance. The present paper describes a protocol used to investigate the problem of (de) synchronisation of biological times (chronotypes) in childhood and youth in relation to school times. It studies the effects of student chronotype vs. school schedule matches/mismatches on health behaviours (e.g., how many hours students sleep, when they sleep, eat, do physical activity, spend time outdoors in daylight) and learning (verbal expression, spatial structuring, operations) and whether alert-fatigue levels mediate this effect alignments/misalignments on learning (verbal expression, spatial structuring, operations) and their mediation by alert-fatigue levels. The novelty of our protocol lies in its multidisciplinary and mixed methodology approach to a relevant and complex issue. It draws on up-to-date knowledge from the areas of biology, medicine, psychology, pedagogy and sociology. The methods employed include a varied repertoire of techniques from hormonal analysis (cortisol and melatonin), continuous activity and light monitoring, self-registration of food intake, sleep timings, exercise and exposure to screens, alongside with systematic application of cognitive performance tests (e.g., memory, reasoning, calculation, attention) and self-reported well-being. This comprehensive and interdisciplinary protocol should support evidence-based education policy measures related to school time organisation. Appropriate and healthier school timetables will contribute to social change, healthier students and with more efficient learning. The results of studies using a similar methodology in other countries would ensure replication and comparability of results and contribute to knowledge to support policy making.

## 1 Introduction

Since the 1920s, it has been found that circadian rhythms influence more than 100 human functions (1). Circadian rhythms are synchronised to the 24-h light/dark cycle (solar time), the most visible circadian rhythm being the sleep-awake cycle. However, people adapt differently to the environment and we call these differences chronotypes. There is a physiological response to light exposure, and a later exposure to light reflects in a later sleep–wake behaviour. Residents in countries located more west of their time zones tend to exhibit a later sleep–wake behaviour (2). The most extreme west country in the Central European Time zone (CET) is Spain (3, 4), which makes its residents more vulnerable to be a late sleep chronotype and, consequently, more vulnerable to circadian misalignment (abnormal timing between different cycles) (5). In fact, as pointed out by Pin et al. (6), 37% of adolescents aged 12 to 15 from the Valencia region (Spain) believe they get little sleep during the week. Higher misalignment between solar time and clock time (solar jetlag) has been associated with shorter sleep duration, especially in late chronotypes (7). The group most affected by sleep loss is adolescents since during this phase of their development they present an endogenous delay in their biological rhythms and a greater difficulty to build sleep pressure (5) resulting in a later sleep onset timing and higher circadian misalignment. A highly used proxy for circadian misalignment is the so called ‘social jetlag’ that is calculated by the difference between the midpoint of sleep on free days and workdays (3). Circadian misalignment can potentially affect health (8, 9) and well-being during adolescence, a life stage characterised by a decline in subjective well-being (10, 11). The potential effects of circadian misalignment on both physical and mental health during adolescence are likely to be reflected by high levels of sleep deprivation or even school absence (12), while a better alignment between chronotype and school timing is associated with lower grade retention in adolescents (13).

As was shown in the 2011 Progress in International Reading Literacy Study (PIRLS) and Trends in International Mathematics and Science Study (TIMSS) a gap in reading ability and mathematics (11 points difference in each case) was associated with sleep deprivation (14, 15). The number of 4th-year (10 year old) students suffering sleep deprivation was 49%, which increases by 10% for 8th grade students (14 years old). Meijer (16) pointed out that chronic reductions in sleep can have direct and indirect negative effects on school performance through the effect on motivation and attention. Several studies show that later school starting times improve students’ sleep (17), performance (18) and life satisfaction (19).

School schedules are the main influence on the organisation of time in the lives of children and youth. They affect how much time they can sleep (1, 16, 20–23). This influence has been already reported in several studies and for different ages (24–27). The consequences of not getting enough rest and going to classes at a time that is out of kilter with their biological clock (during sleep time) affects students’ school performance, with late chronotypes being the most severely affected. Andrade and Menna-Barreto’s (28) study of adolescent girls (16 year olds) showed that they tended to achieve worse scores for tests held in the morning and higher scores for those held in the afternoon. Wolfson and Carskadon (29) studied 3,120 students aged between 13 and 19 and found that students awarded the lowest grades (C, D, and E in the North American system) went to bed 40 min later on average and slept 25 min less a night on average compared to those students who obtained the highest grades (A and B) (30). There is other evidence linking the late chronotype with poorer academic performance (12, 31) and the positive effect on student grades of scheduling exams later in the day and the week. For example, van der Vinne et al. (32) show that the chronotype effect on academic results disappears if exams are scheduled after noon and Pin et al.’s (33) study shows that moving exams from Monday mornings to Wednesday’s mid-morning, increased average scores by 1 point (out of 10). School performance of late chronotypes becomes increasingly worse with age, being worst at high school which can affect future academic career and labour earning opportunities (34). Gromada and Shewbridge (30) found that students attending the morning school schedule but with later preferences on study time present higher levels of absenteeism. Contrary to much debate on school timings, it seems that ‘when’ matters as much or even more than ‘how much’ and, to be inclusive, school timetables would need to be tailored to students’ optimum study times and learning rhythms. Evidence from recent studies in Latin America also show similar effects of the different school timings on adolescents chronotype, sleep and performance (26, 27, 35–37).

Insufficient sleep and being forced to wake in their ‘biological night’ can also affect students’ nutrition through skipping breakfast (38) or being forced to eat during their biological night, which affects insulin sensitivity and increases the risk of diabetes (39). PIRLS 2011 (15) and TIMSS 2011 (14) claim that 27–29% of 4th-year (age 10) students, on average, are affected ‘somewhat or a lot’ by insufficient food intake before certain classes. This has a negative effect on reading achievement and mathematics (14, 15). Evening or late chronotype students are the most disadvantaged; a later chronotype has been linked to a higher probability of obesity and unhealthy behaviours such as smoking, drinking and excess consumption of caffeine (9, 38, 40).

Psychological and bio-psychological studies have been examining the link between the time of the day that a task is performed and performance efficiency. Gabaldón-Estevan and Obiol-Francés (41) point to the consensus about the ultradian oscillation of attention rhythms among primary school students in terms of identification of two alert cycles. Up to noon, attention (alertness) increases followed be a decrease until 1,400 and then another increase which extends into the evening hours. A study by Testu (42) indicates that children aged between 10 and 11 years, show low levels of alertness between 0800 and 0900 and then increased alertness which peaks between 1,100 and noon. After the school lunch break, alertness levels are again low, but then increase and peak around 1,600. Similar alertness patterns have been found to occur among children in the United Kingdom, Germany, Spain, Israel and the United States (30).

How people evaluate their lives, regardless of age—in general and in relation to specific life domains (family, friends, leisure time, etc.)—has been described as Subjective Well-Being (SWB) (43). SWB has both a cognitive (life satisfaction) component and a two dimensional (positive and negative) affective component, which reflects the acknowledged tripartite structure of SWB theory (44–46). In a parallel with SWB, Psychological Well-Being (PWB) is also important for children’s and adolescents’ overall well-being; several scholars suggest that SWB and PWB are complementary concepts within the broader construct of well-being (47–49). The current protocol subscribes to this idea.

In much of the literature, the focus has been either on SWB or PWB, measured using different instruments and different theoretical approaches, depending on the particular philosophical origin [the hedonic tradition focuses on SWB in terms of feelings of pleasure (happiness)]; the eudaimonic tradition focuses on the feeling of happiness derived from feeling fulfilled as an individual. In contrast to SWB instruments, PWB are not differentiated by a more cognitive or affective aspect but by the concrete dimensions they include (e.g., self-acceptance, positive relationships with others, autonomy, etc.).

Most protocols to date are either specific in their scope or limited in their tools. Most studies use either cortisol or melatonin or actigraphy or questionnaires to determine chronotype. Some of them use two or three of these tools, but hardly any of them use the four as in our proposed protocol. Several important studies relate chronotype to academic performance, but not many include student wellbeing with objective measures of aptitude. Finally, the protocol we propose is unique in that it also includes a complete study of the subjects’ time use in connection to all the other tools used. Our proposed protocol should advance research in this area of the School Health Promotion by providing different objective measures (i.e., actimetry, hormonal and cognitive function assessment) as well as subjective measures (i.e., questionnaires, diaries). We consider that our protocol could help future researchers by providing a common measure which will ensure the replicability of our method. We call the protocol Kairos, after the Greek mythological god, because we agree with him that the focus should be on ‘opportunity’ rather than ‘fate’.

**Figure 1:**
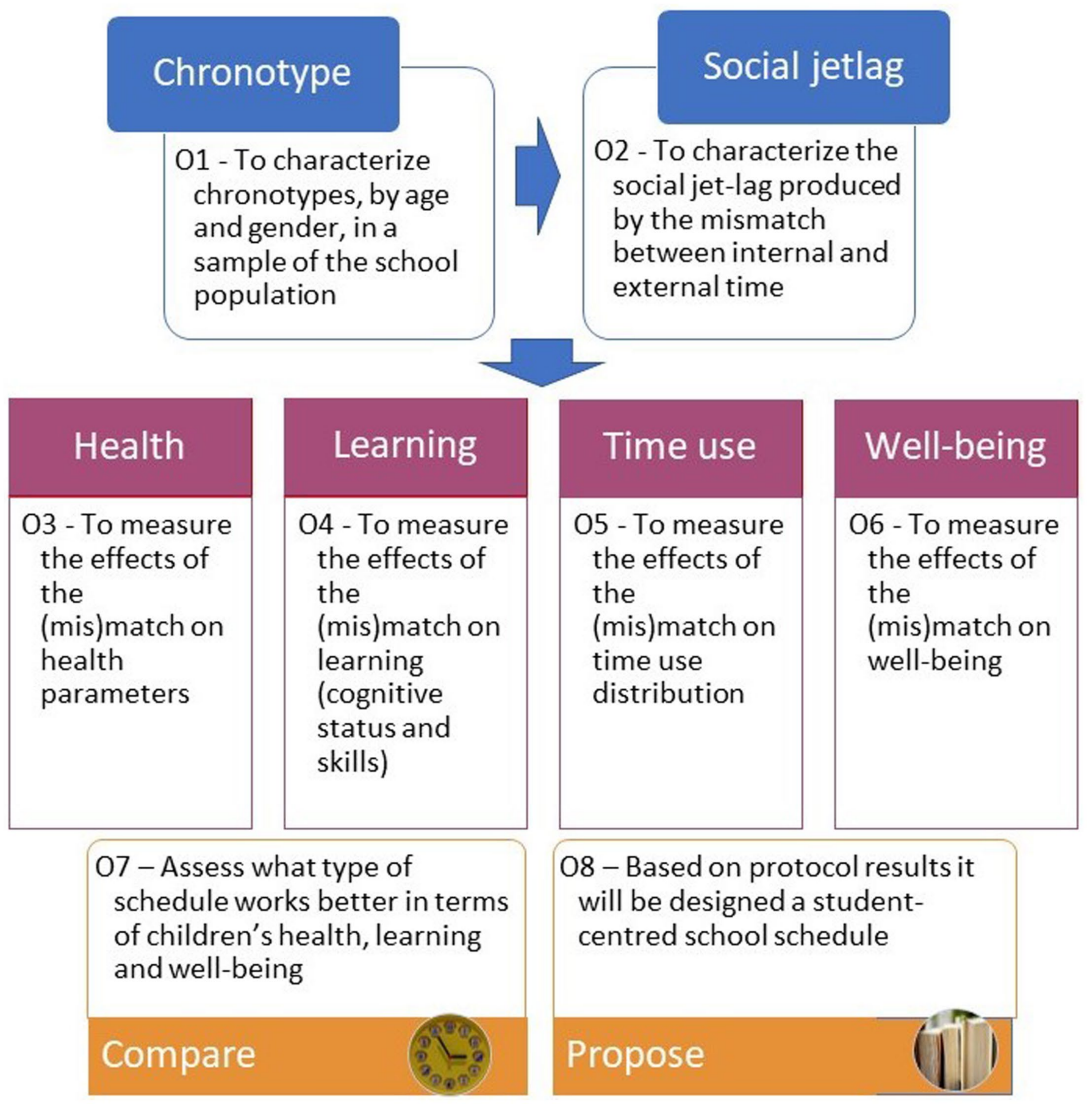
Specific objectives—the Kairos protocol.

The aim is to provide a reference protocol to promote the organisation of school schedules that respect the needs and well-being of children and adolescents. Figure 1 depicts the objectives included in the protocol.

The specific objectives of the Kairos protocol are:

O1—To characterise the chronotypes in the school population based on the distribution of chronotypes across a sample of students and applying objective (cortisol/melatonin in saliva, actigraphy data) and subjective (questionnaire and diary) measures;

O2—To quantify the social jetlag induced by the mismatch between student chronotype and social (school) schedule based on an assessment of the occurrence of social jetlag in a sample of students, measured as the difference between the midsleep point on non-school days (waking up without an alarm) and the midsleep point on school days;

O3—To measure the effect of both chronotype and social jetlag on sleep (length and quality), eating/mealtime patterns (eating time and type of food consumed), active (non-sedentary) time and time spent outside;

O4—To assess the effects of chronotype and social jetlag on cognitive status (vigilance, alertness, attention) and skills (motor coordination, simple calculation, memory tasks);

O5—To measure the effect of both chronotype and social jetlag on the distribution of time use and satisfaction with performance of activities in the student sample, through self-reported distribution of the time devoted to sleeping, meals, playing video games, watching TV/videos (screen time) and doing homework;

O6—To measure the effect of both chronotype and social jetlag on students’ well-being, comprised by SWB (overall life satisfaction, satisfaction with specific life domains, positive affect and negative affect) -thus expanding the traditional tripartite model discussed above with the quadripartite model recently proposed by Savahl et al. (50), and PWB;

O7—To measure the effect of the school schedule on the parameters included in O1 to O6; In the case of education systems (e.g., Colombia, Germany, Italy, Mexico, Spain, etc.) where school schedules may differ within a single education system (e.g., split vs. compact, morning vs. evening, different start and/or break times, etc.), O7 could include the comparison between these; O8—As stated earlier, the ultimate aim of this Protocol is to contribute to the promotion of school schedules that are responsive to, and therefore respectful of, the needs and well-being of children and young people. To facilitate the dissemination of results and promote social change, we encourage the design of student-centred school schedules based on the knowledge gained from the application of the Protocol.

The Kairos Study Protocol proposes a collaborative strategy related to study design and collection and analysis of replicable and comparable data. Section 2 describes the protocol in more detail and Section 3 discusses its pros and cons.

## 2 Methods and analysis

The Kairos protocol is aimed at addressing the problem of (de)-synchronised time experienced by children and youth. We investigate the effects of the social jetlag of students on health (how much sleep, timing of sleep, timing and quantity of food, time spent on physical activity and time spent outdoors in daylight), learning (verbal expression, spatial structuring), mediated by alert/activation and fatigue levels, day-time activities (studying, socialising, time spent with family, time watching screens, etc.), and self-reported well-being. We explore the impact of students’ social jetlag on their lives in terms of health, learning, time use and well-being.

### 2.1 Target sample

Power analysis can be calculated considering the outcome of interest and the statistical test to be used (e.g., T-test, ANOVA). The significance level is set at *p* < 0.05, for a power of 0.80. For our study, the sample was defined as a minimum of 385 measurements/surveys needed to have a 95% confidence level that the true value is within ±5% of the measured/surveyed value. The sample includes school students (6 to 18 years of age), enrolled in primary schools or secondary (high) schools. The students are to be selected on the basis of willingness to participate and agreement from parents or guardians. The intervention demands informing schools, families and participants and communication channels with the parties for queries and to report the general findings to the schools and participants involved. Inclusion criteria are children of both sexes over 6 years of age enrolled in compulsory education, who agree to participate in the study and are enrolled in schools that agree to participate. Exclusion criteria will be not in compulsory education, unable or unwilling to follow the protocol, or not in a school that has agreed to participate.

The most critical aspects are recruitment and respondents’ consistency in following the protocol. To ensure this, it is necessary to prepare information sheets explaining the use of each instrument, to hold information meetings with the centre management, with the teachers of the participating groups, with the students and with the parents of the students. The schools, teachers, students and parents must give their consent for the students to participate. This makes random selection impossible, but to ensure a representative sample, schools are to be selected based on certain criteria including social class of student’s families. The recruitment strategy should be top-down, first obtaining permission from the Ministry of Education to carry out the study, then obtaining the agreement of the school management and class tutors, and only then recruiting families and students. To do this, information letters and information sessions should be made available to potential participants, their families and teachers.

### 2.2 Logistics for participants

The protocol is suited to both cross sectional and longitudinal research designs. For example, in the latter case, the first year’s data obtained from screening 1 will include the full protocol for children aged 6, 9 and 12 and the second year’s data obtained from screening 2 will be applied to the same students will then be aged 7, 10 and 13 years, and so on. This allows for collection of longitudinal data which can be used to propose causality. To compensate for sample dropout, additional individuals who meet the required criteria can be included in subsequent screenings (see figure Flowchart of study population in the Supplementary material for target pursue in Kairos study).

**Figure 2:**
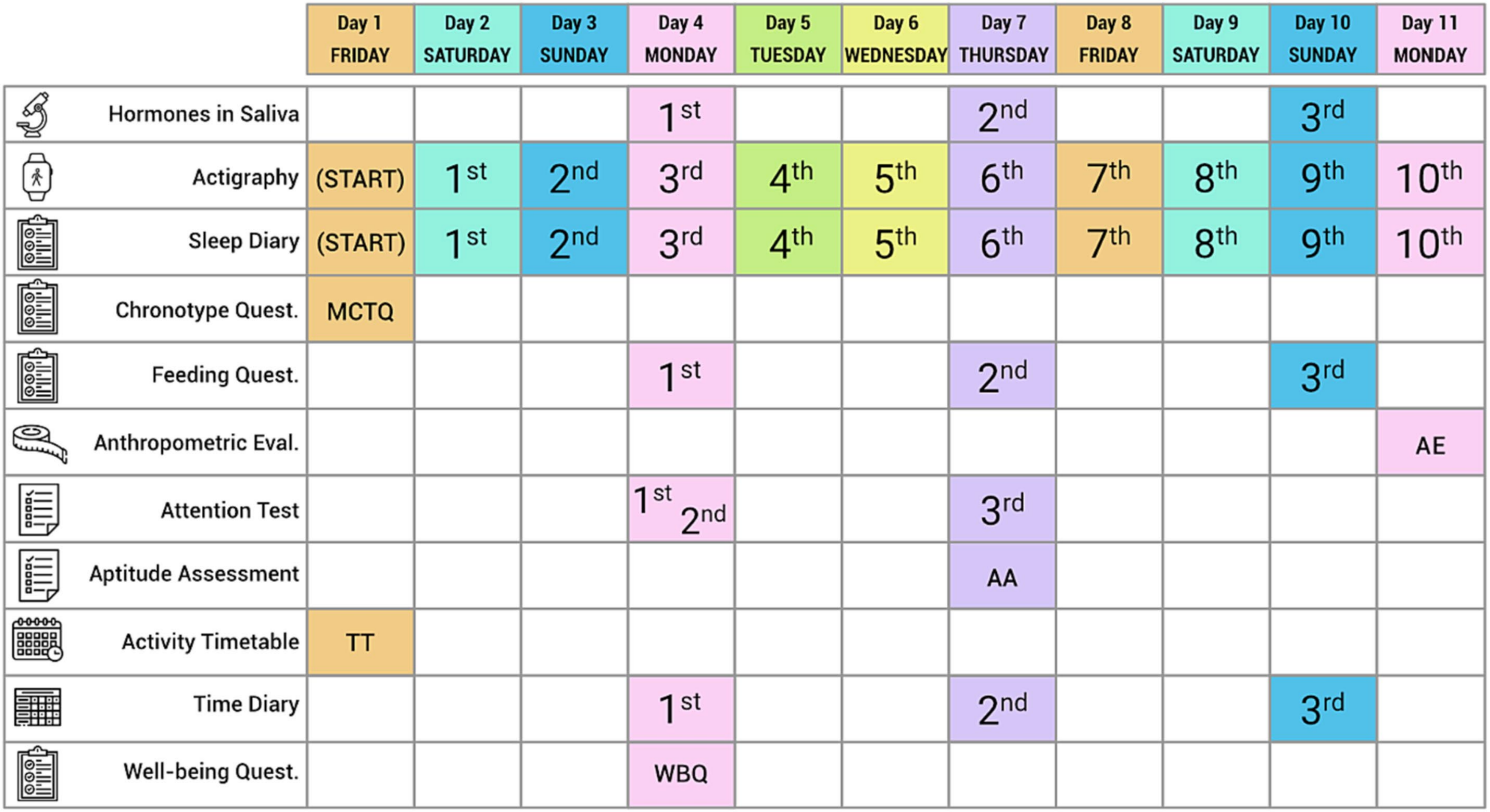
Calendarization of tools.

The model explanatory variables are age, gender, chronotype, social jetlag and school schedule. Our outcome variables are grouped into health (sleep, eating, activity and time out), learning (cognitive: wakefulness, alertness and attention; skills: motor coordination, simple calculations, memory), time use (patterns and satisfaction with activities) and well-being (SWB and PWB). Figure 2 provides an overview of the protocol.

Figure 2 shows that the protocol combines both objective and subjective measures to assess social jetlag and its effects on students’ health, learning, time use and well-being. It uses a total of 11 instruments which capture the complexity of the topic under study. In our biological measures/assessments (saliva, anthropometry), we include actigraphy monitoring (of sleep, temperature, activity and exposure to daylight) along with data derived from the sleep and time use diaries, the cognitive assessment (attention and aptitude) tests and questionnaires (Munich Chronotype Questionnaire—MCTQ, food/eating questionnaire, timetable of activities and well-being questionnaire).

The protocol is designed to be applied over a period of 11 days to allow for five full schooldays plus four full non-school days, to allow to test students’ attention on two different Mondays and on a Thursday. To maintain data confidentiality, students are assigned individual project codes. To allow for follow ups, the school administrations are given the list of project codes assigned to participants and the parent or guardian authorizations. Sociodemographic data and some additional information (school marks, special needs, etc.) is to be obtained directly from the relevant school administration. In the succeeding subsections, we describe the individual instruments.

### 2.3 Instruments

#### 2.3.1 Hormonal assessment based on saliva samples

Previous studies have used the measurement of salivary cortisol to assess the differences between its levels and the correlation of these differences with the subjects’ chronotype (51). Measured salivary cortisol on awakening and 1 h later, on two consecutive days, in 112 subjects who had previously been classified as morning (9 subjects) or evening (29 subjects) chronotypes according to the Horne and Ostberg *Owl-and-Lark-Questionnaire*. They showed that those who identified themselves as morning chronotypes had higher salivary cortisol levels on waking (day 1) or 1 h after waking (day 2) than those who identified themselves as evening chronotypes. Petrowski et al. (52) attempted to replicate the findings of Kudielka et al. (51) in the controlled environment of a sleep laboratory. In this study, 1,023 participants were classified as morning (29 subjects) or evening (59 subjects) chronotypes using the Morningness-Eveningness-Questionnaire (MEQ). Saliva samples were taken on waking and after 15 and 30 min. Their results showed that chronotype influences cortisol levels on awakening and subsequent cortisol levels, which were higher in subjects with a morning chronotype. Weidenauer et al. (53) found similar results, but they also looked at differences between weekdays and weekends. As Kunz-Ebrecht et al. (54) have already observed, cortisol levels are higher on weekdays than on weekends. On the other hand, these studies measured cortisol levels early in the morning without assessing cortisol curves throughout the day. Miller et al. (55) gather several results that suggest that more negative diurnal cortisol slopes correlate with adaptive states of the subjects, while more positive slopes, closer to zero, could indicate altered states of the cortisol axis.

Regarding melatonin measurement Griefahn et al. (56) carried out an hourly measurement for 24-26 h of salivary melatonin in a controlled environment. The results showed that subjects identified with morning chronotype (7 subjects) using the MEQ showed a melatonin secretion profile with maximum concentration about 3 h earlier than subjects with evening chronotype (14 subjects). Another study with a very similar design with 33 subjects was carried out by Liu et al. (57). The results of melatonin measurements every hour for 26 h also showed that subjects with morning chronotype according to the MEQ have the melatonin peak earlier than those with evening chronotype.

The same results were obtained by Lack et al. (10), showing 2–3 h earlier in the peak of melatonin for subjects with morning chronotype. In this protocol, subjects also underwent 27 h of monitoring and measurement of cortisol every hour. In our study, we will use the information discussed above and the recommendation of Pandi-Perumal et al. (58) on the indications for obtaining partial melatonin curves. As the approximate time at which the maximum peak of melatonin and Dim Light Melatonin Onset (DLMO) is obtained for both subjects with morning and evening chronotype is known, we will reduce the intervention on the subjects since it is not feasible to perform 24-26 h cortisol curves. In addition, we will carry out the measurements on three different days of the week, with the aim of looking for possible differences, as occurs in the case of cortisol.

**Figure 3:**
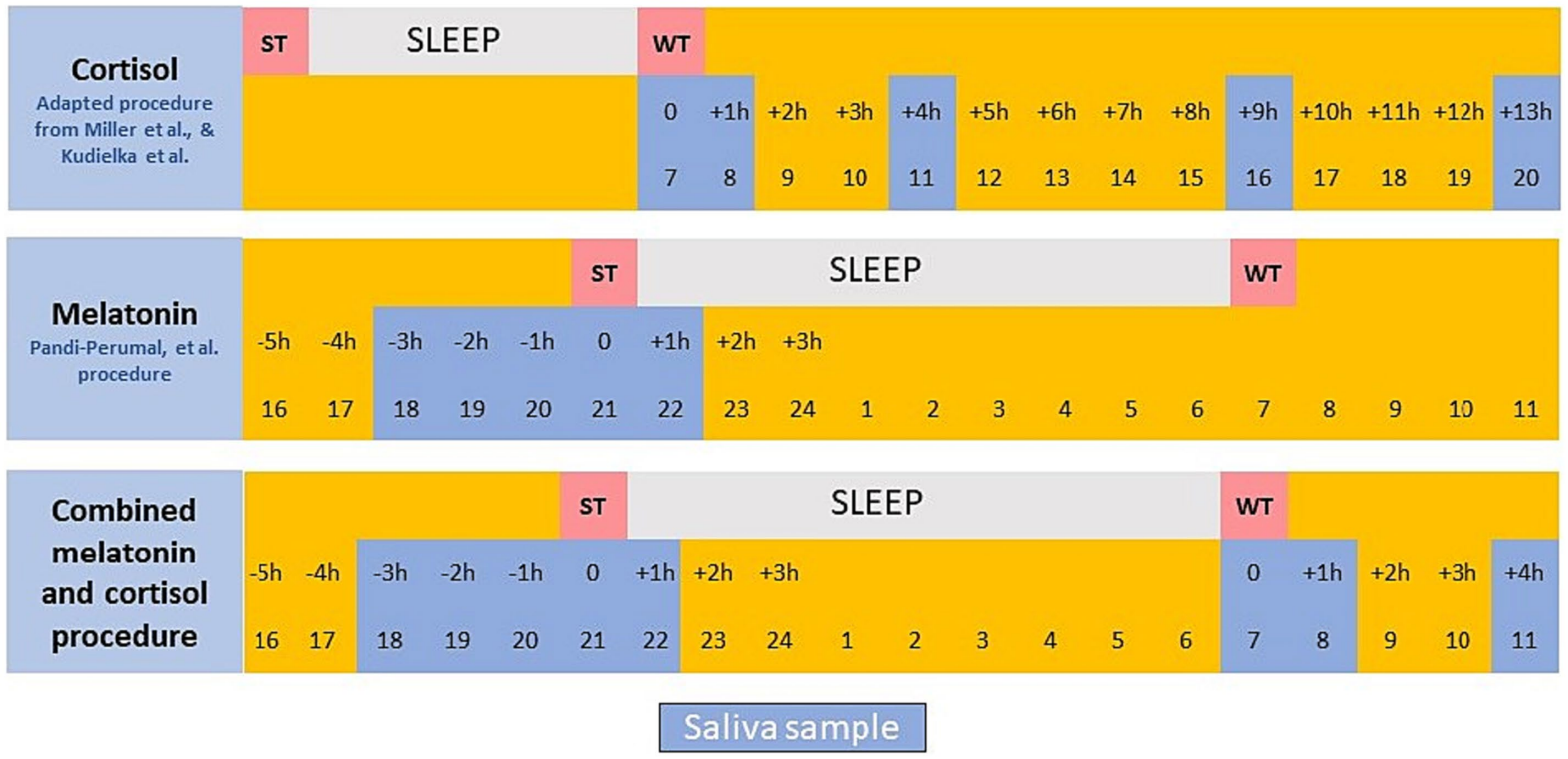
Saliva sampling procedure.

Saliva samples are collected to measure Dim Light Melatonin Onset (DLMO) and cortisol peak. To obtain the daily curve for cortisol the samples will be collected at 0, 1, 4, 9and 13 h after waking (51, 55). The minimum saliva DLMO threshold is 4 pg./mL (59) and the recommended partial melatonin curve to calculate DLMO should include values between at least 19 and 23 h (probable delayed sleep phase) or between 16 and 21 h (probable advanced sleep phase) (58), for a minimum of five samples in total (60). The relationship between DLMO and sleep onset timing is on average ~ 2 h before sleep onset (61, 62), although this value may vary substantially (e.g., DLMO ranging from ~4.5 h before to 0.5 h after sleep onset time) (62), especially for late chronotypes (63, 64). As so, the ideal collection timing for hourly saliva collections, would start 5 h before sleep onset time and ends until 1-2 h after sleep onset time. However, if having budget restrictions, we may reduce sample collections until 3 h before habitual sleep onset and goes until 1 h after habitual sleep onset time in order to comply with the 5 timepoints for DLMO determination. This allows for a combined salivary cortisol and melatonin collection procedure based on 8 collection times: sleep time-3, sleep time-2, sleep time-1, sleep time, sleep time + 1, awake time, awake time + 1 and awake time + 4 (see Figure 3). Cortisol and melatonin are measured at the same time at 20 (sleep time-1) hours; the remaining sampling is hormone-specific and takes place during execution of daily activities. It will be analysed the maximum cortisol levels first thing in the morning, the decline curve of cortisol during the day and the DLMO. The interpretation of these cortisol and melatonin curves will provide important information about the chronotype of the subjects and allow their classification according to Kudielka et al. (51) and Petrowski et al. (52). In addition, they will allow to delve deeper into participants state of health as, for example, a more negative slope of cortisol is associated with better health—according to Miller et al. (55)—and an altered DLMO is associated with circadian imbalances and sleep and mood disorders—according to Pandi-Perumal et al. (58).

If the budget does not allow assessment of melatonin, the protocol applied only to cortisol provides the daily curve for cortisol and establishes the better-fit saliva curve collection protocol for the standard cortisol pattern. The sampling (collection of saliva in tubes) should take place during execution of daily activities.

To reduce the level of intervention for the students, we suggest that sampling should be confined to Monday, Thursday and Sunday of the same week (see Figure 1). This will allow assessment of the evolution of cortisol and melatonin levels through a week and, also, differences across different days.

It is recommended that saliva is collected in Sarsted Salivette Cortisol^®^ tubes, following their sampling protocol. The students should have not taken in any liquid or solid food (including chewing gum) for at least 60 min before the samples are taken and must rinse their mouths with water 10 min before sampling. The evening saliva collections should be performed under dim light (< 5lux). The swab should remain in the mouth for 2 min to allow for maximum possible sample volume. The tube must record the time of collection and the final sample should be refrigerated as soon as possible after sampling. Processing of the sample should be as per the tube manufacturer’s instructions. Samples are centrifuged for 2 min at 1000 g. and the swab then discarded safely. The sample can then be frozen (but never refrozen) before the cortisol and melatonin concentrations are determined, which requires that the samples are completely thawed and homogenised. Determination of salivary cortisol is performed using the Roche Cobas 6,000^®^ and electrochemiluminescence technology. Salivary melatonin is determined using the DRG-SLV-4779 DRG^®^ Melatonin direct (Salivary) ELISA (Enzyme-Linked ImmunoSorbent Assay) kit; the plate is read using the Grifols Triturus^®^ analyser.

Saliva sampling is non-invasive and is performed by the student (study subjects); it does not involve blood samples or any other clinical intervention. In all cases, students or tutors will be informed verbally and through a detailed written protocol with pictograms about the appropriate collection of the sample. In addition, sample collection during school hours will be supervised by the research group. Finally, they will be instructed to remain seated or inactive as much as possible and to collect saliva samples to measure melatonin in dim light. The study protocol described above has been approved by the Dr. Peset Hospital Bioethics Committee, Valencia (Spain).

Regarding the interpretation of the results: Axelsson et al. (65) and Kudielka et al. (51) showed that early morning cortisol levels were higher in subjects with morning chronotype compared to subjects with afternoon chronotype. Furthermore, several studies summarised in Miller et al. (55) indicated that a greater (negative) slope of cortisol decline during the day is indicative of subjects’ adaptation, whereas flatter slopes of cortisol decline could indicate deregulation of the axis. Therefore, interpreting the curves with five daily cortisol points on three different days will allow to know the patients’ chronotype more accurately. On the one hand, the concentration of cortisol on waking or the maximum cortisol level is measured first thing in the morning, which allows to distinguish between the morning and evening chronotype of each subject. On the other hand, the slope of the curve will be studied to assess the health status of the subjects and to differentiate those with alterations in the cortisol secretion axis that may be caused, for example, by stress. Furthermore, comparing this information between the continuous shift and split shift groups can provide information that allows us to assess which type of shift is most beneficial for the subjects. The data obtained will provide the students’ salivary cortisol and DLMO and morning melatonin values and allow them to be linked to their health, stress and sleep quality states. It will show how these hormone levels are influenced by or influence the other instruments (see Discussion section).

#### 2.3.2 Actigraphy

Actigraphy is used to record the body rhythms that are controlled by the circadian system (66, 67). These rhythms are used as markers and provide information on the working of the circadian system. These rhythms are influenced, also, by other variables, such as light and sleep, so we recommend recording more than one rhythm simultaneously. To allow correct measurements and result, these rhythms must be monitored over several full days with high sampling frequency, to reduce the variability inherent in individual lifestyles. Alongside salivary secretion of melatonin and cortisol, the most frequent rhythms are related to physical activity and body temperature and are monitored using actigraphy. Actigraphy is a convenient and cost-efficient method to record (activity-inactivity, temperature, and light exposure, body position) over multiple days in a natural environment. It is especially useful for samples that include infants and young children whose sleep is likely to be affected by a laboratory environment (68–70).

The participants follow their usual routines at home and in their school environment. Their activity is sampled at 10 Hz and stored in every 30 s epochs. Measurements taken when the actimetry watch is not being worn are excluded from the analysis and if in 1 day we have more than 4 h of missing data that day is eliminated for analysis. We calculate sleep onset, sleep offset and sleep duration for schooldays and non-school days according to Madrid-Navarro et al. (71). We also measure nap behaviour since many adolescents nap to make up for lack of sleep at night; this is an indirect indicator of how tired they are, and the total sleep time should be estimated for the 24 h period. All individuals involved in the actimetry test will be required to use the event marker of the device to provide the following information: sleep onset and sleep offset times.

To record circadian rhythms and sleep, we use the actigraphy device Kronowise Feedback^®^ multichannel device, which allows simultaneous recording of skin temperature rhythms, physical activity, position, light exposure (infrared, blue and total visible light) and sleep over a prolonged time period, allowing, in turn, evaluation of appearance of chronobiological changes (including sleep changes) and follow-up over a full week.

The Kronowise Feedback^®^ device (Kronohealth SL, Spain) includes: 1. a temperature sensor with precision ±0.1°C at 25°C and 0.0635°C resolution, housed in a separate chamber to avoid thermal interference from the battery and electronic components, and attached to a metal plate that is in contact with the skin; 2. a triaxial MEMS (Micro ElectroMechanical System) accelerometer calibrated with linear and equal sensitivity along all three axes, within the range ± 2 g. and sensitivity 0.001 g.; 3. 50/60 Hz.

The device records approximately 23 million raw data points related to 14 primary variables and 1 estimated variable (sleep) that reflect the functioning of the circadian system. The information obtained includes: (1) daily physical rhythms [integrated variable TAPL—rist skin temperature (T), motor activity (A), body position (P) and light exposure (L)] (72–74); (2) sleep parameters such as sleep latency, total sleep time, counts of Waking After Sleep Onset (WASO), sleep efficiency, activity during sleep and the timing of sleep (onset, offset and mid-time); (3) data to allow calculation of the main indicators of circadian health (regularity, contrast between day and night, synchronisation and quality of rest).

The main circadian indexes will be determined by non-parametric analysis, including:

- VM5: average value of the variable during the 5 consecutive hours of maximum values. It applies to variables that increase during sleep, such as temperature and sleep;
- HM5: central time of the 5 consecutive hours of maximum values. Values that increase during rest periods;
- VL10: Average value of the variable during the 10 consecutive hours of minimum values. Variables such as temperature and sleep, whose values decrease during awake periods;
- HL10: central time of the 10 consecutive hours of minimum values. Values, such as temperature and sleep, that decrease during wake periods;
- VM10: average value of the variable during the 10 consecutive hours of maximum values. Variables such as motor activity, position and light exposure, whose values increase during awake periods;
- HM10: central time of the 10 consecutive hours of maximum values, for values, such as motor activity, position and light exposure, that increase during awake periods;
- VL5: average value of the variable during the 5 consecutive hours of minimum values in relation to those variables, such as motor activity, position and light exposure, whose values decrease during sleep;
- HL5: central time of the 5 consecutive hours of minimum values. Values such as motor activity, position and light exposure, that decrease during rest periods;
- IS (Interdaily Stability): a score of the regularity of circadian pattern over different days. This varies between 0 for a Gaussian noise to 1 for a total stability, where the rhythm repeats itself exactly, day after day, we assess it for skin temperature, physical activity, position, light exposure (infrared, blue and total visible light) and sleep;
- RA (Relative Amplitude): for variables that increase during resting times or during activity times, we assess it for skin temperature, physical activity, position, light exposure (infrared, blue and total visible light) and sleep;
- ES (Environmental Synchronisation): degree of synchronisation between the phase marker of a circadian variable during a rest phase (HM5 or HL5) and the centre of natural darkness taking as the reference the Central European Daylight Time (CEDT) and the location where the event is registered. This variable can express values between 0 and 1. When the centre of a variable coincides with the centre of natural darkness, ES is 1.
- CHS or CHI (Circadian Healthy Score/Circadian Healthy Index): is the main marker of circadian health. Its values range between 0 and 1. The value 1 indicates a rhythm perfectly synchronised with the natural light–dark cycle in terms of regularity, environmental synchronisation and normalised relative amplitude, as described by Martínez Nicolas et al. (75). This value is used as a global marker of chronodisruption: the closer to 1, the less chronodisruption.

All in all, we justify the use of actigraphy because it gives us the opportunity to study the person over a long period of time in their usual environment, clearly identifying moments of activity and rest, and it is also a small, comfortable and portable device (like a watch). Also, because it provides objective data that we can compare with data obtained from other instruments in the protocol (triangulation). Finally, it allows us to assess the person’s exposure to light, which allows us to make a good assessment of possible circadian problems, and, by measuring temperature, we can find out whether the person’s time to go to sleep coincides with their biological time for doing so.

#### 2.3.3 Sleep diary

The student or tutor must record what time the sleep ‘ritual’ begins, what time the individual estimates to fall asleep and what time the student wakes up. Daytime naps and any events that occur during the night must be registered. Physical activity and use of technology must also be registered. These data should be registered over a 10-day period when the student is wearing the actigraph (i.e., school days and non-schooldays). Those completing the sleep diaries will be the subjects themselves in the case of secondary school students, and their parents or guardians in the case of primary school students. Information sessions will be held for both groups and members of the research team will be available to answer any questions.

#### 2.3.4 Munich chronotype questionnaire

The core version of the standard MCTQ (Children and Adolescents version) is used to assess sleep-awake behaviour on school days and non-schooldays. The MCTQ asks about sleep and activity times for schooldays and for school-free days: what time the individual goes to bed; what time he or she decides to go to sleep; how long it takes to fall asleep; what time the individual wakes up, what time the student gets up and if an alarm clock is used. The MCTQ is a validated widely used method to estimate chronotype, based on the midpoint between sleep onset and sleep termination on school-free days, ‘corrected’ for sleep debt accumulated across schooldays; this provides the chronotype estimation in the form of a local time not a score [for the exact calculation, see (3)]. Additional variables, such as sleep duration and social jetlag (absolute difference between mid-sleep on non-school and school days, i.e., the difference between biological and social time) can be retrieved from the MCTQ. Chronotype and social jetlag calculations are considered only if the participant reports not using an alarm clock on non-school days. Additional measures enabled by the MCTQ are sleep latency, sleep onset and wake up times, sleep midpoint, time in bed, and sleep duration separately for school-and non-school days.

Finally, we also include the item “You hear about ‘morning’ and ‘evening’ types of people. Which of these types do you think you are?” with four possible answers because it is an item from the Morningness-Eveningness Questionnaire, which is widely used as a single item to reflect circadian self-perception.

#### 2.3.5 Eating and food questionnaire

Assessment of food intake and timing is required to understand eating patterns and the contribution of macro and micronutrients at the individual and collective levels. The dietary questionnaire allows an assessment of food intake. To estimate the individual’s usual intake, the respondent is asked to record all food and drink taken at each meal on a Monday and a Thursday (schooldays) and one non-school day, Sunday. Prior to the start of the study, the researcher will provide instructions, an example and three templates for these records to both the participant and an adult member of the household who will support the student. The document asks about: (1) amount of food ingested; (2) all the ingredients in a particular meal; (3) the cooking method; (4) location of the meal (if at school, attach the school meal menu); timing of the meal; (5) drinks; (6) snacks taken between meals; and (7) whether the food and drinks were homemade or processed.

These data allow two calculations. First, the nutritional composition of the student’s diet can be measured, based on the dietary record that can be calculated using a software such as DIAL (Alce Ingeniería SA Madrid, Spain). The most relevant diet variables are kilocalories consumed, percentages of proteins, carbohydrates and lipids, amounts of sugars, total, soluble and insoluble dietary fibre, cholesterol, and saturated, monounsaturated and polyunsaturated fatty acids. Second, data on chrononutrition and the timing of intake on school days and non-school days. The tool is very easy to fill out because it is a description of what they eat. For elementary school students, it is completed by their parents, and for high school students, by themselves. Our experience with the tool so far suggests that it is an easy task for participants to complete if they are reminded to do so. Studies suggest that recording daily food intake and calculating calories using food exchange lists and food composition tables to estimate caloric intake are within an acceptable range of error and therefore represent a practical approach to estimating caloric intake (76).

#### 2.3.6 Anthropometric evaluation

Anthropometric evaluation is used to record the following health parameters weight and height, body mass index, body composition, skin folds, waist circumference and is applied to the whole sample. Anthropometric evaluation has been widely use in the literature for body composition analysis (77, 78). For this protocol participants must be weighed and measured, by the same person, under the same conditions in line with International Society for the Advancement of Kinanthropometry (ISAK) (79) recommendations. A bioelectronic scale with a body composition analyser should be used to record body weight and the other body composition parameters. This is a non-invasive method to allow evaluation of body composition by measuring the opposition to the flow of a current through the subject’s body. More resistance (greater bioimpedance) will be linked to individuals with large amounts of adipose tissue which is a poor conductor of electricity due to its low water content. Bioimpedance allows estimation of total body water and fat-free mass and fat mass segmented by body areas. Accurate measurement of bioelectrical impedance requires the student to have urinated immediately before, be dressed only in underwear, with bare feet, with nothing metallic on their person or clothing, standing upright with arms outstretched. Privacy should be maintained by the use of a 3-leaf paraban or similar.

A stadiometer with 0.1 cm fraction precision should be use for the height measurements. Students should be bare foot and standing with their feet together, with their heels, buttocks, back and occipital region in contact with the vertical plane of the stadiometer, with the feet together. When taking the reading, the student must inhale and keep his or her head in line with the Frankfort plane (imaginary horizontal line between the lower edge of the orbit and the external auditory canal). We recommend that height should be recorded in centimetres.

The height and weight data allow calculation of the z-BMI score and the corresponding percentiles using the SEGHNP2 Nutritional Application programme and the reference tables for the focal country’s paediatric population (in our case Spain) to calculate the percentile and the equivalent SD for each subject. A child with a BMI greater than or equal to 1.8 standard deviation (SD) above the mean for age and sex is classed as obese, according to the World Health Organisation (WHO) (80). The students’ waist should be measured manually using a non-extensible, flexible steel tape calibrated in centimetres with millimetre graduations in line with ISAK (79) protocols. The waist measurement should be taken by placing the tape 4 centimetres above the navel with the student standing upright and breathing normally. The waist measurement is recorded at the end of an outbreath. Tricipital, bicipital and abdominal skin folds should also be measured to allow calculation of body fat. Three measurements must be taken of the waist circumference to obtain the average among the values. Three measurements of each fold should be recorded by the same person and under ISAK (79) titration.

#### 2.3.7 Attention test

Attention tests allow estimation of optimal attention time slots to enable optimal student learning. The tests should be administered to each student three times to compare results at the beginning and end of the first school-day in the week (Monday—1st and last class session) and to compare those with mid-morning Thursday. Different attention tests have been chosen to avoid a training effect derived from repetition of the same test within a few days. We recommend that the Monday 1st class tests should use the Test of Perception of Differences (or FACES-R test) and Monday end of the day tests should use the d2-R and the Thursday attention test should be based on attention sub-test included in the Aptitude Assessment test (see Section 3.7).

The Faces-R test is used to evaluate perceptual and attentional aspects among subjects aged between 6 and 18 years old and is appropriate for large samples. It comprises 60 graphic elements, each grouped according to three schematic faces, two of which are identical and a third which has one different feature (mouth, eyebrow, hair, etc.). The d2-R test is a timed test, suitable for subjects aged 6 years and older and involves a cancellation task to evaluate selective attention. It measures processing speed, ability to follow instructions and accuracy of execution of a task involving discrimination among similar visual stimuli. Both tests provide scores for speed and accuracy, which are important aspects of the main score—ability to concentrate.

To observe and record attention changes throughout the day and the week among a group of students requires administration of the attention test at different times and on different days. We recommend three tests: first and last classes on Monday and mid-morning on Thursday. This allows observation of attentiveness and activity at the beginning and end of the first school day in the week (Monday) which can be compared with attentiveness and activity in the middle of the day on the second to last day of the week (Thursday). The results of these tests will indicate the hours when level of attention is highest, which will favour understanding and internalisation of new learning, and those days in the week when perception is perhaps highest. Both tests have been used in other protocols with the same population target (81–84).

The test is conducted in the same way for the whole sample, regardless of age. First, the test should be distributed to each individual in the sample; the subjects will be asked to complete the information requested on the first page (identification data). The psychologist or pedagogist then will explain the test. After ensuring all the study subjects have understood what is required the test should start. The time allocated to completing the test is 3 min.

After 3 min, the test pages will be collected. The responses will be awarded direct scores, percentile scores or STANdard NINes (stanines). Higher scores indicate higher capacity for attention and perception and greater control of impulsivity.

Internal consistency of the FACES-R test will be established using Cronbach’s Alpha coefficient; we obtained a score of 0.91 for the whole sample. However, since the Cronbach’s Alpha coefficient is below 0.90 for students aged between 6 and 11 years, we need to apply some additional instruments to increase reliability.

Based on the results obtained from summing hits and errors, the following profiles can be identified.

The results of this test should be correlated with the results of the aptitude tests such as BADyG (Bateria de Actividades mentales Diferenciales y Generales or Differential and General Skills Battery; see next instrument) used to detect aptitude levels of sample participants. BADyG also includes an attention subtest that will be used as the attention test for Thursday. It is useful, also, to cross-check these data against student chronotype, extracted from the MCTQ, to verify whether the hours of peak attention correspond to the individual’s periods of peak energy and activity.

#### 2.3.8 Aptitude assessment

Aptitude is a construct that cannot be observed directly; therefore, aptitude should be measured using a self-reported questionnaire. The BADyG aptitude test allows the assessment of academic performance and special educational needs and has been widely used in research (85, 86). There are different versions of the BADyG test to suit ages 4 to 18 years. The test is copyright protected and must be purchased by the researcher. The test shows good psychometric scores, excellent reliability at all levels and good internal consistency of Cronbach’s alpha, Spearman-Brown coefficient and Guttman’s two halves coefficient. The BADyG is used to measures cognitive development in verbal, numerical, spatial and logical reasoning skills and ability to solve verbal, numerical and spatial syllogisms, and the speed and efficiency with which students solve the academic problems they encounter. It also provides a score for general intelligence.

BADyG logical reasoning includes inductive operations and abstract concepts based on grouping visuospatial, numerical and verbal aspects. It requires ability to solve comprehension problems and access to memory. The logical reasoning score is obtained by summing the scores for analogue relations, numerical series and logical matrices. The verbal score is obtained by combining the content of verbal compression and analogical reasoning to obtain second-order relationships between pairs of concepts. This ability is basic and central to most smart activities. The verbal score also involves Analogue Relations plus Complete Sentences tests. The numerical part of the test requires access to memory and recovery of previously learned knowledge, decoding of numerical codes or symbols and the related operations. The numerical score is obtained from the Numerical Series plus Numerical Problems test. The visuospatial test involves representations of figures that rotate a series of degrees to left or right which must be positioned appropriately to complete the painting. This is like Raven’s Progressive Matrices, which are used as a non-verbal measure of overall intelligence and are obtained from the Logical Matrices + Fit figures. General intelligence is considered to combine all mental abilities: the mind’s relational and abstractive activities. However, it is current intelligence, not something given at and immutable since birth. It is based on development phases and interaction with environmental stimuli and is obtained by summing the verbal analogies, numerical series, logical matrices, complete sentences, numerical problems and figure fitting scores.

The tests should be scheduled to cover a range of times appropriate to the student’s age: for example, older aged students will require less time to complete the test. For a sample with an average age of 12 years, the basic tests will take 67 min and the complementary tests 24 min, to a total of 91 min. It should be noted that within each level of the various tests, the questions are organised in order of difficulty, from easier to more difficult. Correct application of these tests requires subjects to have a test book and another book in which to record their answers. Each test must be explained to participants along with the time available to complete the test. A computerised version of the tests is also available.

Interpretation of the results is done based on individual answer books/sheets.

The results allow automatic generation of reports. The collective report is generated by the direct scores and the group centile score, which provide scaled and statistical results. Individual profiles are obtained by comparing a specific student’s percentile with the percentile assigned to each test scale. The comparative intergroup report of means is extracted by comparing the mean of each group with that of the corresponding scale; the means of each test are then compared to allow observation of those skills that are more developed and those that require interventions. The intragroup level distribution report is based on the percentile ranking in the 7 levels: a score of 94 or over is Very High, a score of between 93 and 75 is High, 74 to 61 is Medium-High, 60 to 31 is Medium, 30 to 16 is Medium-Low level, 15 to 7 is Low and the score of less than 7 is Very Low level. Finally, the interval between general intelligence and intelligence quotient or IQ indicates the upper and lower limits of student percentile scores.

One of the limitations of the BADyG and similar tests, is the length of time involved and the need for some breaks during the tests to avoid a fatigue effect which could lead to lower test scores. We recommend a 30-min break halfway through the battery of tests.

#### 2.3.9 Timetable of activities

The student timetable for a normal week captures their participation in a range of different activities (school hours, extracurricular activities, sports trainings, etc.). It requires them to indicate start and finish times for their regular activities and provides an understanding of how primary and secondary school students allocate their time, on a weekly basis. Also, and in line with Csíkszentmihályi’s (87) ‘flow’ concept, students are asked to rank their level of preparedness (skill) for the individual activities (low, medium or high skill level) and how difficult (low, medium or high) they found the activity. Measuring the ‘flow’ related to each activity captures the student’s subjective evaluation of his or her routine activities.

#### 2.3.10 Time diary

The Time Diary is used is to measure the student’s participation in different activities based on information on time spent on learning, socialising, leisure, self-care and other activities. The Time Diary tool asks participants to indicate their main and secondary activities, who is involved in these activities and where they take place, on a scale of 10-minute slots, allowing a degree of objectification of non-or partially-institutionalised social activities and their duration (88). The Time Diary from the standard Time Use Survey (TUS) provides a detailed record of the activities undertaken during one school day and one non-school day. The TUS is administered in numerous countries and, in the European Union, is coordinated by the Eurostat Working Party which devised the Harmonised European Time-use Study (HETUS) in the late 1990s (89).

In the case of primary age children and adolescents, the interest is in obtaining information on students’ daily routines, with whom they interact and their assessed level of satisfaction with these activities. To respond to the specificities of the study subjects and the research objectives, the TUS Time Diary can be adjusted to include these aspects.

Students are asked to record their activities over three specific days, with support from tutors, parents or guardians. Using a 10-min interval scale, the students are asked to indicate their activities over the course of two school days (Monday and Tuesday) and one non-school day (Sunday). Figure 1 shows that these 3 days coincide with the collection of saliva samples, recording of eating in the feeding questionnaire, wearing the actimetry watches and recording their sleep/awake behaviours in the sleep diary. This pattern facilitates data collection, and the range of data allows for data triangulation.

#### 2.3.11 Well-being questionnaire

The literature recommends the use of more than one type of instrument to allow comparison and allow collection of complementary information (90). The wellbeing questionnaire uses the five scales employed in the third wave of the Children’s Worlds Project^1^ to assess SWB and PWB. The CW-SWBS, CW-DBSWBS, CW-PNAS and CW-PSWBS are adapted from previously proposed scales. It also includes four additional satisfactions with life domains not being part of any scale. The limitations regarding cross-cultural comparison are discussed later. All of the instruments are answered under a self-application form and only for students 8-years-old and older. They are the following:

##### 2.3.11.1 Overall life satisfaction scale

The importance of a single item scale for overall satisfaction in the context of the cognitive dimension of SWB was highlighted by Campbell et al. (43). Although initially formulated for adults, this scale has been used widely with samples of children and adolescents and has shown good performance. We included a question asking about overall satisfaction with life; 10 to 18 year-old students are asked to indicate their overall satisfaction with their lives on a scale from 0 to 10, where 0 is Not at all satisfied and 10 “Totally satisfied” and a scale of 5 emoticons (from sad to happy) for 8 to 9 year olds. The questions posed are: for 10 to 18-year-old students—To *what extent are you satisfied with your life in general*? And for 8- to 9-year-old students—*Are you happy with your life in general*?

##### 2.3.11.2 The children’s worlds subjective well-being scale

The CW-SWBS consists of six items measuring cognitive SWB, based on Huebner’s (91) Student Life Satisfaction Scale, Diener et al.’s (92) Satisfaction With Life Scale, and suggestions made by the children themselves on how to improve the wording. They are the following: *I have a good life, The things that happen in my life are excellent, I am happy with my life, I enjoy my life, My life is going well and I like my life*. An 11-point agreement scale was is used from 0 = Not at all agree to 10 = Totally agree (10 to 18 year olds), and an agreement 5-point scale ranging from “I do not agree” to “Totally agree” (8 to 9 year olds). An overall index is calculated based on summing the six items and dividing the total by 6.

##### 2.3.11.3 The children’s worlds domain based subjective well-being scale

The CW-DBSWBS includes five items measuring domain based cognitive SWB, based on Seligson et al.’s (93) Brief Multidimensional Student Life Satisfaction Scale (BMSLSS). The five items refer to satisfaction with: *The people you live with; Your friends; Your life as a student; The area you live in;* and *How you look*. The CW-DBSWBS is scored on an 11-point scale from 0 = Not at all satisfied to 10 = Totally satisfied for 10- to18-year-old students and a scale of 5 emoticons (from sad to happy) for primary 8- to 9-year-olds school students. Calculation of an overall index is achieved by summing the five items and dividing the total by 5. Again, there are some limitations in relation to cross-cultural comparison and these are discussed later.

Besides the CW-DBSWBS, the protocol also includes additional satisfaction with life domains not being part of any scale but also included in the Children’s Worlds project to cover more aspects of children’s and adolescents’ lives. They are measured in the same way as for the CW-DBSWBS and are analysed separately. They are satisfaction with how you use your time, the freedom you have, your health and how are you listened to by adults in general.

##### 2.3.11.4 The children’s worlds positive and negative affects scale

The CW-PNAS measures the affective dimension of SWB and is based on the Core Affect Scale (94, 95). The six items include three for positive affect (*full of energy, happy, calm*) and three for negative affect (*stressed, sad, bored*). The question posed for 10–18-year-old students is: Please consider each of these words and then indicate on a scale from 0 (*Not at all*) to 10 (*Extremely*) how much you felt this way during the last 2 weeks? while 8–9-year-olds students are asked to indicate frequency of these feelings on a 4-point scale ranging from “*Never*” to “*Always*,” with “*Sometimes*” and “*Often*” as intermediate options. An overall index can be calculated by summing the three items on positive affect and the three items on negative affect and dividing the respective scores by 3. The items can also be analysed separately.

##### 2.3.11.5 The children’s worlds psychological subjective well-being scale

The six-item CW-PSWB psychometric scale measures PWB and is based on Ryff’s (96) model [see (97)]. They are asked to score the following items: I *like being the way I am; I am good at managing my daily responsibilities; People are generally friendly towards me; I have enough freedom of choice about how I spend my time; I feel that I am learning a lot at the moment;* and *I feel positive about my future* on an 11-point unipolar scale from 0 “Totally disagree” to 10 “Totally agree.” This scale is only administered to the secondary-education students (12 to 18 year olds). An overall index summing up the six items on positive affect, on the one hand, and negative affect, on the other hand, and dividing the respective total scores by six is generally calculated.

Casas and González-Carrasco (98) investigated the reliability and comparability of the CW-SWBS, CW-DBSWBS, CW-PNAS and CW-PSWBS among the 35 countries included in the third wave of the Children’s Worlds Project, based on the 10- to 12-year-old age groups. In the CW-SWBS, they suggested that an abridged version of the five items, which excluded the item *I like my life*, would be suitable for both 10 and 12 year olds. Comparison of the data from the different countries based on multi-group equation modelling showed that the mean scores for the item *The things that happen in my life are excellent* was not strictly comparable among countries for either age group, although the other items were satisfactory. The five items in the CW-DBSWBS were deemed appropriate for both age groups. However, the authors warned against use of a statistic based on an overall index for cross country comparison, since the meaning differs across countries.

For both age groups, the explained variance among the CW-DBSWBS items may be smaller compared to the CW-SWBS items. For the pooled sample, the CW-PNAS (positive and negative affect) models are appropriate for both the 10-year-old and 12-year-old samples. The multiple-group model is appropriate for the 10-year-olds, but not the 12-year-old group. Again, a statistic based on an overall index for the scale should not be used for cross country comparison. The CW-PSWBS is appropriate for both age groups, but again no statistic based on an overall index should be used for cross country comparison. For the CW-DBSWBS, CW-PNAS and CW-PSWBS cross country comparison can be based on correlations and regressions. For the primary age students, confirmatory factor analysis of the CW-SWBS applied to the 8-year-olds group, corresponding to the third wave of the Children’s Worlds Project showed adequate fit for structural equation modelling while the multi-group analyses supported scalar invariance if one of the countries was excluded from the model. Therefore, findings support application of the CW-SWBS for 8 year olds in both eastern and western countries (99).

We need to make some comments on the interpretation of the results of the above instruments. In general terms it could be said that the higher the score the better. However, there is a range of values that are likely to define most of the sample population’s responses to the SWB (there is no equivalence for the PWB level). The theory behind the predicted normative range for the SWB scores is Cummins (100) homeostatic model. To explain variations in SWB we suggest the analogy of deviations in blood pressure and heart rate, which, in certain circumstances, due perhaps to different external and external factors (e.g., stress, illness) exhibit minimum and maximum values, and which under normal circumstances, return to their baseline values.

It is assumed that there is a genetic mechanism that controls SWB homeostatically. There may be relatively small variations in SWB levels among individuals belonging to the same culture which can be explained by this mechanism, although with the exception of cases where inbuilt protection mechanisms fail. The range of values for adults of between 75 and 90 on a 100-point scale, is due to optimism bias (100, 101) whereas the normal distribution is around 50. Although some authors suggest that levels of SWB are similar for younger children and adolescents (102), there is some evidence suggesting that these set-points are higher among primary age children and decrease through adolescence before converging, at some point, with the adult population (10, 11).

Taking the third wave of the Children’s Worlds project and the only scale that supports mean comparisons across countries, the five-item version of the CW-SWBS, as a reference point, this decreasing with age tendency reported by González-Carrasco et al. (10, 11) with longitudinal data is observed for the group of 10-year-olds compared to 12-year-olds (the same scale is used for both). The mean score for the 8-year-olds ranges from 3.11 to 3.65 out of 4 (with Spain ranked 3rd—mean of 3.59). The means for the 10-year-olds group oscillates between 7.94 and 9.71 out of 10 (with Spain ranked 4th position). And the means in the case of the 12-year-olds group (mean of 8.82 out of 10) with means ranging from 7.25 to 9.55 (with Spain ranked 7th position) (103).

### 2.4 Ethics and dissemination

The protocol complies with Spanish legislation (Biomedical Research Law, BOE July 4, 2007, research collecting data on humans), and the ethical standards articulated in the Helsinki declaration (104). All the processes involved comply with international ethics of human genetic studies recommendations (105). Participation carries no risks for participants; it involves no invasive procedures and no interventions with known risky side effects. The saliva samples are used only to collect hormonal indicators.

The data (salivary hormones, actigraphy data, subjective data collected through questionnaires and diaries, personal data including age, sex, socioeconomic background, etc.) are collected in line with the guidelines set by an Ethics Committee. Use of the personal data collected during application of the protocol should be limited to project fieldwork and is subject to the participants’ informed consent. Personal identity will be protected through use of anonymized codes; only the schools and high schools involved will be able to related the codes to actual names. The codes will never be made public. The documentation and work data will be stored on secure servers to which only the research team will have access.

At the end of the project, the anonymized data, will be made available to the scientific community in line with national legislation. During the project the members of the research team will not process data using personal means and will guarantee security through conformance to legal requirements acknowledging that resources containing personal data are confidential and restricted. At the end of the project, any personal data collected will be destroyed.

### 2.5 Data analysis

The protocol generates a considerable flow of data, which should be recorded, organised, and stored in a huge set of quantitative and qualitative variables of different nature. The preliminary analysis should include the initial treatment of records, data cleaning, and graphical representations. Then, marginal and multivariate analysis of cross-sectional variables of interest should be performed by using diverse descriptive and association measures, multiple regression models, binary choice models, and in the cases of repeated measurement, panel data methods.

A key assumption in linear regression is that observational units (e.g., students) are independent, given the values of a set of covariates. An important violation of the independence assumption is due to clustered data, when the responses of students are naturally grouped in classrooms, or schools. The analyst could never hope to observe all the potentially influential covariates, such as the classroom atmosphere, the teacher’s enthusiasm and competence, or the level of parental involvement, since they cannot be measured appropriately. Therefore, there is unobserved heterogeneity between the responses by classrooms and schools. The consequence is that two observations in same classroom or school are correlated and more similar than observations in different classrooms or schools, so students in one classroom or school would tend to have better test results, even after controlling for observed covariates, than students in another classroom or school. Multilevel models allow the processing of data from hierarchical structures and are especially useful for data processing in educational research. This analysis complements the previous ones, so that it considers not only the possibility of treating the first-level elements that characterise individual responses, but also the different levels of nested models that can be proposed, considering higher-level effects than the individual student.

## 3 Discussion

Epidemiological studies show that chrono disruption is a risk factor for numerous diseases, including cardiovascular disease, metabolic syndrome, cognitive and affective disorders, cancer and immunosuppression, among others (106, 107). Exposure to light and physical or mental activity at hours when exposure to light is abnormal for human beings causes alterations to biological rhythms and, especially, sleep rhythms and biological processes controlled by endogenous circadian clocks. These effects are seen particularly among children and adolescents. The use of electronic technology at the end of the school day and during extracurricular and family activities contribute to tiredness during daytime hours and are contributing factors for school failure (108).

Exposure to artificial light (in school, or at home) leads to changes to clock genes and proteins in the central nervous system and affects melatonin and other hormones production, which affect the duration and quality of sleep and changes to central and peripheral circadian rhythms (109, 110). Activities such as mealtimes and the type of food consumed also affect peripheral clocks which in turn affect circadian rhythms (111).

There are studies that it exemplifies some of the effects of the school schedule in the health, performance and wellbeing of students. Some studies link delayed intake of food with increased obesity (8, 40) also in children and youth (112) and some highlight the link between compact schedule in school with decreased performance and increased fatigue (113–115). Several works focus on the effects of school time organisation in schools and its relationship with (de)-synchronisation with students’ internal body clocks (12, 31, 32). With the effect of school schedule organisation on academic performance (13, 116) and the impact of school schedules on sleep (23, 26, 117).

The protocol captures several health measures to evaluate the effect of school schedules on youth health and wellbeing. The protocol measures circadian health, amount and quality of sleep, body mass index, body composition, diet, outdoor light exposure, and minutes per day of moderate-to-vigorous physical activity, as well as recreational screen time. As for wellbeing questionnaire, as we already mentioned, we integrate the five scales employed in the third wave of the Children’s Worlds Project and include four additional satisfactions with life domains not being part of any scale.

The protocol allows the comparison of different school schedules (extended vs. compact; morning vs. evening) within an education system. We propose to measure the effects of different school schedules on students’ health, wellbeing and academic performance by controlling for the other confounding variables included in the protocol. Although demanding, compliance with the protocol is possible with a reasonable degree of success if the research team carries out its tasks of field preparation, information, support and instrument collection properly. We believe that the more support given to students and parents in explaining the purpose of the research and how the protocol works, the greater the success. In some cases, a second appointment to retake a test or complete a questionnaire must be granted if the participating student is ill or unable to attend, and if this happens, it must be on the same day of the week and at the same time as the original request.

From a scientific-technical perspective, the combination of expertise and methodologies proposed in this protocol is novel and should be of value to both researchers and education institutions. In 2023, we implemented the protocol in two high schools with 49 13-year-old students. The protocol included measuring cortisol levels, but not melatonin due to budget limitations. This prior experience has enabled us to adjust logistical aspects of the protocol based on our field study. The protocol enables the collection of rich and complementary data from a sample of students, which will provide a better understanding of the complexities related to the scheduling of lesson times for children and adolescents. The statistical analysis should include both a longitudinal description of each variable and a multivariate description of the relationships between them based on extensive correlation analysis. The ability to attribute causality, which is rare in this field, is enabled by collection of longitudinal data, which also enable a better mapping of the changes that occur in childhood and adolescence. For example, the proposed protocol allows collection of data on children’s cortisol levels, during two different days during the school week and on a non-school day. These data related to young people are unique.

Our protocol allows the interlinking and further development of several research strands (on sleep, eating, activity, learning, socialisation, well-being and other patterns) within a comprehensive, interdisciplinary and complex protocol. The findings will reinforce existing research and should be informative for policies in education and aspects related to school timings. In turn, this should contribute to the formulation of more healthy, efficient and satisfying school schedules. The novelty of our protocol should contribute to research and education worldwide and the well-being of society and, especially, students, students’ families and teachers. These benefits may contribute be manifested in better student performance in school and a better school climate and improved health literacy.

We are aware of the inherent challenges in achieving full synchronisation between the school timetable and individual chronotypes, and we are also aware that getting the school timetable right for students’ chronotypes is only part of the problem, but a very important one. What happens outside of school is very relevant to what happens inside, and the pedagogy and internal organisation of academic activities also play an important role in both student performance and well-being. We believe that both what happens outside school and what happens inside school will benefit greatly from a better match between the school timetable and the chronotype of the student. On the other hand, there is a degree of scepticism about improving school schedules due to the multifactorial nature of the educational process, especially in education systems with a weak tradition of evidence-based practise. We believe that it is only through the development of research and specific educational studies that we will be able to guide teachers, families and policy makers in a research-action process of social change.

Considering the protocol and the accompanying instruments, the following strengths and weaknesses are identified. At the application level, one of the protocol’s strengths is its multidisciplinary approach, encompassing various disciplines and variables to study a broad age range, with a sample ranging from 6 to 16 years, considering each developmental and academic stage. On the flip side, one of the most critical aspects is the recruitment and consistency of the sample requiring participants to commit to the protocol with up to 12 instruments applied over 10 days. While it is a strength that applied as a longitudinal study allows the evaluation of the evolution of many variables over time, maintaining the sample for several years poses a significant challenge. To compensate for sample dropout, additional individuals who meet the required criteria can be included in subsequent screenings. Additionally, as a weakness, reliance on the sample’s responsibility in completing instruments is acknowledged, with increased completeness when applied in the school setting assisted by the class teacher and/or the research team.

On the instrument level, strengths include obtaining hormones through non-invasive saliva samples and the actigraphy as a wearable instrument capable of registering extensive data over an extended period in student’s natural environment. The feed questionnaire, attention tests, and well-being questionnaire stand out for their ease of application, speed, applicability across a wide age range, and provision of valuable data for student contextualisation. The time diary with its 10-min interval scale allows triangulation both the actimetry and the sleep diary facilitating the assessment of data consistency. One of the cons is that the BADyG test requires a significant amount of time for application, leading to decreased student attention due to fatigue towards the end if a break is not schedule.

## Ethics statement

The studies involving humans have been approved by Dr. Peset Hospital Bioethics Committee, Valencia (Spain). The studies will be conducted in accordance with the local legislation and institutional requirements. Written informed consent will be obtained from the participants and the participants’ legal guardian/next of kin prior to participation.

## Author contributions

DG-E: Conceptualization, Funding acquisition, Methodology, Supervision, Writing – original draft, Writing – review & editing, Project administration, Visualization. DC-T: Methodology, Visualization, Writing – original draft. BC-G: Methodology, Writing – original draft. EM-G: Methodology, Writing – original draft. VM-C: Methodology, Writing – original draft. LM: Methodology, Writing – original draft. EM-B: Methodology, Writing – original draft. MG-C: Methodology, Writing – original draft. MH-J: Writing – review & editing. KT: Supervision, Writing – review & editing. MT: Writing – review & editing. AA-A: Writing – review & editing. GS: Supervision, Writing – review & editing. NE: Supervision, Writing – review & editing. GP-A: Supervision, Writing – review & editing, Conceptualization. CR: Methodology, Supervision, Writing – original draft, Writing – review & editing, Conceptualization.

## Footnotes

^1^ https://isciweb.org
