# Supplementary figures and images for: Kairos study protocol: a multidisciplinary approach to the study of school timing and its effects on health, well-being and students’ performance
Source: Front Public Health. 2024 Mar 8;12:1336028. doi: 10.3389/fpubh.2024.1336028 (PMC10957785; doi:10.3389/fpubh.2024.1336028)

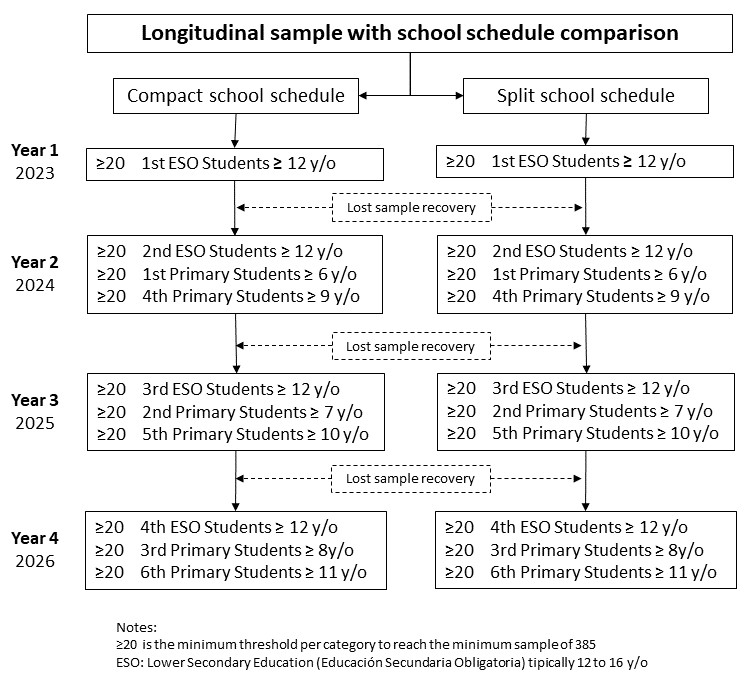

Supplement: Supplementary file 1 [file Image_1.JPEG]
